# P-891. Clinical Impact of Central Venous Catheter-Only Positive Cultures in *Staphylococcus aureus* Bacteremia

**DOI:** 10.1093/ofid/ofae631.1082

**Published:** 2025-01-29

**Authors:** Choseok Yoon, Euijin Chang, Seongman Bae, Jiwon Jung, Min Jae Kim, Yong Pil Chong, Sang-Oh Lee, Sang-Ho Choi, Sung-Han Kim, Yang Soo Kim

**Affiliations:** Hanyang University Seoul Hosptial, Seoul, Seoul-t'ukpyolsi, Republic of Korea; Asan medical center/Department of Infectious disease, Seoul, Seoul-t'ukpyolsi, Republic of Korea; Asan medical center/Department of Infectious disease, Seoul, Seoul-t'ukpyolsi, Republic of Korea; Asan Medical Center, Seoul, Seoul-t'ukpyolsi, Republic of Korea; Asan Medical Center, Seoul, Seoul-t'ukpyolsi, Republic of Korea; Asan Medical Center, Seoul, Seoul-t'ukpyolsi, Republic of Korea; Asan Medical Center, Seoul, Seoul-t'ukpyolsi, Republic of Korea; Asan Medical Center, Seoul, Seoul-t'ukpyolsi, Republic of Korea; Asan medical center, Seoul, Seoul-t'ukpyolsi, Republic of Korea; Asan Medical Center, Seoul, Seoul-t'ukpyolsi, Republic of Korea

## Abstract

**Background:**

There is limited data on patients with indwelling central venous catheter (CVC) presenting with *Staphylococcus aureus* bacteremia (SAB) isolated exclusively from the CVC, without positive results from peripheral venipuncture. The aim of this study was to assess the clinical characteristics and prognoses of SAB patients with cultures positive only from CVC-drawn samples.

**Figure:**
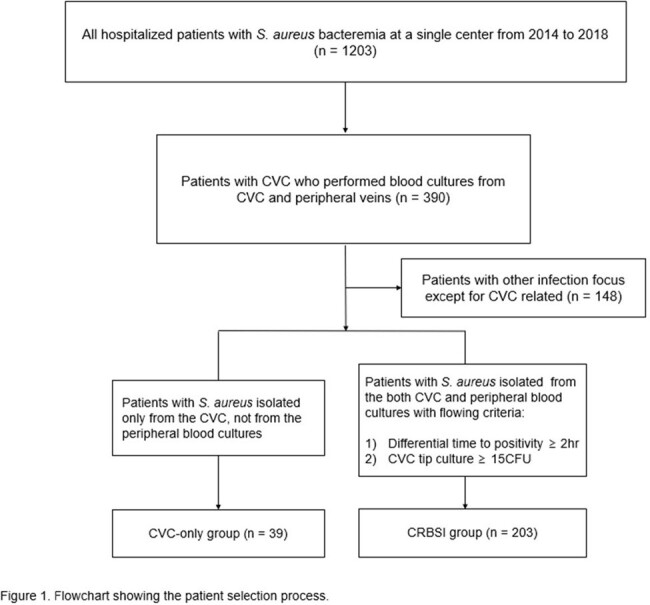

**Methods:**

A retrospective cohort study was conducted from 2014 to 2018 on SAB patients with indwelling CVCs. The prevalence of patients with *S. aureus* isolated only from the CVC (CVC-only positive group) was calculated among SAB patients with indwelling CVCs. The clinical characteristics and 90-day clinical outcomes were compared between CVC-only positive group and those with catheter-related bloodstream infections (CRBSI), where *S. aureus* was positive in peripheral venipuncture as CVC-drawn culture.

**Figure:**
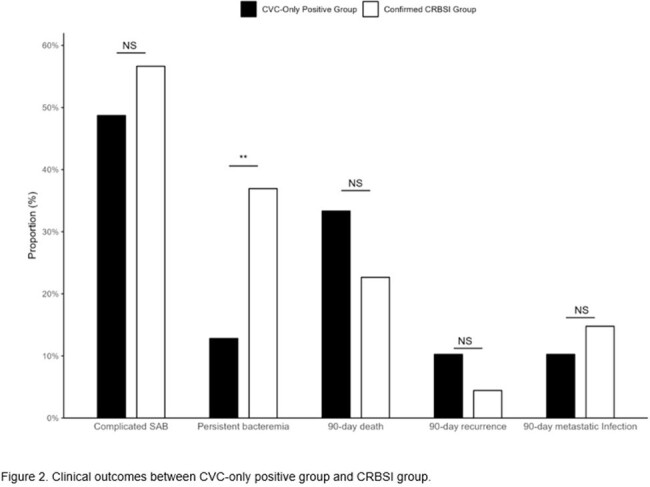

**Results:**

A total of 390 SAB patients with indwelling CVC were identified during 5 years: 39 (10.0%) had cultures positive solely from the CVC, 203 (52.1%) were diagnosed with CRBSI, and the remaining 148 (37.9%) had an infection focus other than the CVC. No significant differences in underlying comorbidities or infection severity of CVC-only positive group compared to the CRBSI group. There were no significant differences in the 90-day outcomes for mortality (33.3% vs. 22.7%), recurrence (10.3% vs. 4.4%), or metastatic infection (10.3% vs. 14.8%) between the CVC-only positive group and the CRBSI group (all P >0.05), except for a significantly lower incidence of persistent bacteremia in the former group (12.8% vs. 36.9%, P=0.006). In multivariable Cox regression analyses adjusted confounders, no significant differences in 90-day mortality (adjusted hazard ratio [aHR] 0.96; 95% confidence interval [CI], 0.50–1.84) or recurrence rates (aHR 1.68; 95% CI, 0.48–5.95) in CVC-only positive group compared to the CRBSI group.

**Figure:**
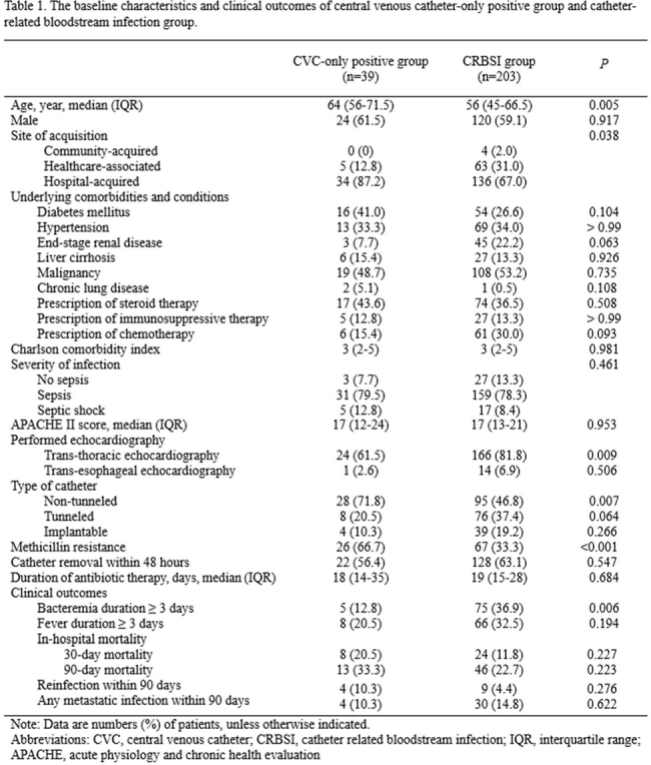

**Conclusion:**

*S. aureus* bacteremia isolated solely from CVCs demonstrated significant mortality and recurrence rates comparable to those observed in CRBSI. These findings indicate that *S. aureus* bacteremia, even when isolated solely from CVCs, should not be dismissed as contamination, underscoring the necessity for appropriate antibiotic treatment.

**Disclosures:**

**All Authors**: No reported disclosures

